# Cytokine-independent induction of LGP2/DHX58 in viral infection

**DOI:** 10.1099/jgv.0.002173

**Published:** 2025-10-31

**Authors:** Yaxin Liu, Xiaohan Tong, Ruixue Wang, Z. Galvin Li, Zichen Xie, Dang Wang, Weikuan Gu, Kui Li

**Affiliations:** 1 Department of Microbiology, Immunology and Biochemistry, University of Tennessee Health Science Center, Memphis, TN 38163, USA; 2 Department of Orthopaedic Surgery and Biomedical Engineering, University of Tennessee Health Science Center, Memphis, TN 38163, USA; 3 Department of Respiratory and Critical Care Medicine, The Second Affiliated Hospital of Harbin Medical University, Harbin, Heilongjiang, PR China; 4 College of Medicine, University of Tennessee Health Science Center, Memphis, TN 38163, USA

**Keywords:** IFN, IRF3, LGP2, NFκB, promoter, RIG-I-like receptor, virus

## Abstract

Laboratory of genetics and physiology 2 (LGP2, also known as DHX58) is unique among members of the RIG-I-like receptor (RLR) family as it lacks the caspase activation and recruitment domain. Although LGP2 per se cannot directly activate downstream antiviral signalling, it plays important regulatory roles, primarily by modulating innate immune responses mediated by RIG-I and MDA5. However, the detailed mechanisms by which LGP2 is induced in mammalian cells during viral infection remain incompletely understood. Herein, we show that LGP2 is strongly upregulated by dsRNA stimulation or virus infection in cultured human cell lines via TLR3 and RLRs, respectively, and that substantial induction of LGP2 remains when paracrine/autocrine signalling of IFNs and/or inflammatory cytokines is abrogated by genetic deletion or chemical inhibition. The latter observation is in stark contrast to the case of myxovirus resistance proteins, the induction of which is strictly IFN-dependent. Mechanistically, we found LGP2 expression to be upregulated by ectopic expression of IRF3-5D, a phospho-mimetic mutant of activated IRF3, or to a lesser extent, by overexpression of RELA, the p65 subunit of NFκB, in an IFN-independent fashion. Additionally, we demonstrated that this regulation operated transcriptionally at the *LGP2* promoter level. Altogether, a fraction of LGP2 induction in viral infection is IFN- and cytokine-independent, highlighting exquisite gene expression control in antiviral innate immunity and representing an evolutionary advantage, which ensures uninterrupted supply of this RLR member protein in host responses to invading viruses in the event that IFN production and/or signalling is disabled by viral means.

## Introduction

Pattern recognition receptors (PRRs) play a critical role in host innate immune responses by detecting pathogen-associated molecular patterns (PAMPs) [1,3]. Among these, the retinoic acid-inducible gene I (RIG-I)-like receptors (RLRs) including RIG-I, melanoma differentiation-associated gene 5 (MDA5) and laboratory of genetics and physiology 2 (LGP2, a.k.a., DHX58) are key sensors of viral infections that engage viral RNAs in the cytoplasm [4,5]. RIG-I and MDA5 activate antiviral responses through their caspase activation and recruitment domains (CARDs), which interact with their adaptor, mitochondrial antiviral signalling (MAVS) protein to trigger downstream activation of transcription factors, IFN-regulatory factor-3 (IRF3) and NFκB, leading to synthesis of type I and type III IFNs [3,6, 7]. In contrast, LGP2 lacks a CARD domain and hence cannot directly activate MAVS. Nevertheless, LGP2 has been shown to coordinate antiviral signalling through RIG-I and MDA5, influencing IFN production [8,10].

The role of LGP2 in viral infection is not fully understood, nor is its own regulation. Studies indicate that LGP2 is crucial for IFN induction during infections by viruses that are sensed by MDA5, such as encephalomyocarditis virus, hepatitis C virus and hepatitis D virus [8,11,13]. LGP2 facilitates the interaction of MDA5 with RNAs and promotes MDA5 filament formation [14]. Its role in RIG-I signalling, however, is complex and, in some cases, virus-specific. Depending on experimental conditions, both positive and negative roles of LGP2 in RIG-I-mediated antiviral responses have been reported [8,13, 15]. While present at low basal levels in uninfected cells, LGP2 mRNA expression is induced after virus infection or following stimulation by IFNs [16,17]. Precisely in mammalian cells how transcription of LGP2 is regulated during viral infection and whether a mechanism independent of IFN autocrine/paracrine signalling – as demonstrated for MDA5 and RIG-I [18,19] – operates, remain unclear.

When mammalian cells respond to viral infections, the secreted IFNs rapidly stimulate the expression of hundreds of IFN-stimulated genes (ISGs), the products of which act collectively to establish a cellular antiviral state. This process, in general, requires autocrine and/or paracrine signalling via engagement of cognate IFN receptors on cell surface followed by activation of the intracellular Jak-STAT pathway [3,20]. The expression of certain ISGs, such as the MX dynamin-like GTPases (MX1 and MX2), is strictly dependent on the IFN autocrine/paracrine feedback loop [21,22]. However, activated IRF3 can directly control the transcription of a handful of ISGs, e.g. ISG56 (a.k.a., IFIT1), ISG54 (a.k.a., IFIT2), zinc-finger antiviral protein, etc., in the absence of the IFN feedback mechanism [23,25]. Based on sequence homology and receptor usage, the IFN family of antiviral cytokines consists of three types: type I (mainly IFN-*β* and IFN-*α*), type II (IFN-*γ*) and type III (IFN-*λ*s). While IFN-*γ* production is restricted to lymphocyte-derived cell types, type I and type III IFNs can be induced in most cell types [3,20, 26]. Given that HeLa cells inherently do not respond to type III IFNs due to lack of the IFN-*λ* receptor IFNLR1 [27], HeLa cells with deletion of the type I IFN receptor IFNAR1 [28] (referred to as IFNAR1-KO) serve as an excellent model to probe IFN-independent expression of specific ISGs in response to viral challenge. In addition, HeLa cells harbour all three viral dsRNA-sensing pathways – Toll-like receptor 3 (TLR3, recognizing extracellular dsRNA), RIG-I and MDA5 (sensing cytoplasmic dsRNA) – that impart robust antiviral gene expression and, as such, have been widely used to study factors that regulate these innate immune mechanisms [6,29, 30]. In this study, we set out to determine the detailed mechanisms of human LGP2 induction during viral infection using HeLa cells with and without IFNAR1 deletion. Combining the use of non-neoplastic hepatocytes treated with and without a pan-JAK inhibitor that blocks IFN and cytokine signalling, we show that while IFN’s autocrine/paracrine action contributes to maximal induction of this RLR member, a substantial fraction of the LGP2 upregulation in response to viral insults is IFN- and cytokine-independent. Mechanistically, we demonstrate that IRF3 and NF*κ*B each play a part in driving LGP2 transcription from its promoter in the absence of IFN autocrine/paracrine signalling.

## Methods

### Cell culture and reagents

Wild-type HeLa (HeLa-WT) and its derived IFNAR1-KO cells (HeLa-IFNAR1-KO) [28] were generous gifts from Gideon Schreiber (Weizmann Institute of Science). PH5CH8 is a Simian virus 40 T antigen-transformed, non-neoplastic human hepatocyte cell line [31,32]. Human embryonic kidney HEK293 (ATCC) and HEK293A (a gift from Jun Hee Lee, University of Michigan) cells were also used in this study. All cell lines were maintained in Dulbecco’s modified Eagle’s medium supplemented with 10% heat-inactivated FBS, 100 units/ml penicillin and streptomycin at 37 °C in a humidified, 5% CO_2_ incubator. Poly-I:C and recombinant human IFN-*α* were purchased from Sigma. Ruxolitinib and caffeic acid phenethyl ester (CAPE) were from LC Laboratories and Cayman Chemical, respectively. Recombinant human IFN-λ1 was from Cell Signaling Technology. Sendai virus (SeV, Cantell strain) stocks were purchased from Charles River Laboratories.

### Plasmids

To construct a luciferase reporter plasmid for studying transcriptional control of human LGP2 gene, a DNA fragment spanning nt positions –888 to +31 of human LGP2 promoter was amplified by PCR from HeLa genomic DNA and inserted between SacI and NheI restriction sites of the promoter-less and enhancer-less pGL3-basic plasmid vector (Promega). The resultant reporter construct was designated pGL3-hLGP2. pGL4-MX1 was a gift from Jane Trepel (National Cancer Institute) and contained a ~0.54 kb long, human MX1 promoter fragment upstream of the firefly luciferase reporter gene [33]. pRL-TK (Promega) was used in co-transfections to normalize transfection efficiencies in reporter gene assays. The cDNA expression constructs for GFP-IRF3-5D [34] (a gift from Nancy Reich, Stony Brook University) and GFP-RELA [35] (a gift from Allan Brasier, University of Wisconsin-Madison) have been described. An expression vector encoding GFP alone (Clontech) was used as a negative control in overexpression experiments.

### RNA interference

Synthetic siRNAs were transfected into cells using Lipofectamine 2000 (Invitrogen) according to manufacturer’s suggested protocol. The IRF3 siRNA (CCCAGGAAGACAUUCUGGAUGAGUU) was from Invitrogen (Catalogue# IRF3HSS105507). A Silencer Negative Control siRNA that does not target any gene product was purchased from Ambion (Catalogue# AM4635).

### Quantitative reverse transcription-PCR

Total RNA was extracted from cells following indicated treatments with TRIzol reagent (Invitrogen) and reverse-transcribed into cDNA using random primers and a standard molecular biology protocol. The relative expression of mRNAs for genes of interest was then determined by quantitative PCR using gene-specific primers, an Applied Biosystems SYBR green master mix and a QuantStudio^®^ 3 Real-Time PCR System. The PCR primers for RIG-I, IFIT3, MX1, IRF3, TRAF1, SeV phosphoprotein and 28S ribosomal RNA have been described [36,38]. The LGP2 primers were: 5′-GAGTACCAGGCCAAGATCCG-3′ (forward) and 5′-CCACCATGCAGTTGATGCAG-3′ (reverse). The relative expression of each target was normalized to that of 28S housekeeping control, and changes in expression between mock and stimulated groups were calculated using the 2−ΔΔCT method [39].

### Immunoblotting

Cellular extracts were prepared, quantified and subjected to SDS-PAGE and immunoblotting as previously described [40]. The following monoclonal (mAb) or polyclonal (pAb) antibodies were used for immunodetection: rabbit anti-LGP2 and anti-MDA5 pAbs (Proteintech); rabbit anti-IFIT1 pAb [41]; rabbit anti-RELB pAb (Santa Cruz Biotechnology); mouse anti-IFIT3, anti-MX1/2/3 and anti-RIG-I mAbs (Santa Cruz Biotechnology); mouse anti-GAPDH mAb (Proteintech); mouse anti-ACTIN mAb (ABclonal); and appropriate IRDye®-labelled secondary antibodies – goat anti-rabbit IgG IRDye® 800CW or goat anti-mouse IgG IRDye® 680RD (both from LI-COR Biosciences). Protein bands were visualized with an Odyssey infrared imaging system (LI-COR Biosciences), and their signal intensity was quantified using Image Studio Lite (LI-COR Biosciences) and normalized to that of a housekeeping loading control (GAPDH or ACTIN).

### Reporter gene assay of promoter activity

Dual-luciferase reporter assay was implemented according to previously described procedures [24,40] to determine the activities of human LGP2 and MX1 promoters in transfected cells following various treatments as specified.

### Statistical analyses

All experiments were carried out with multiple independent replicates, with data expressed as mean±sd. Statistical analyses were performed in R or SPSS, and statistical significance was assessed using Student’s t-test or Welch’s t-test as appropriate. A *P* value of <0.05 was deemed statistically significant. Figures were plotted with ggplot2 or GraphPad Prism 8.

## Results

### Expression of LGP2 is substantially upregulated in both HeLa-WT and HeLa-IFNAR1-KO cells by dsRNA or virus via various PRR pathways

**Fig. 1.**
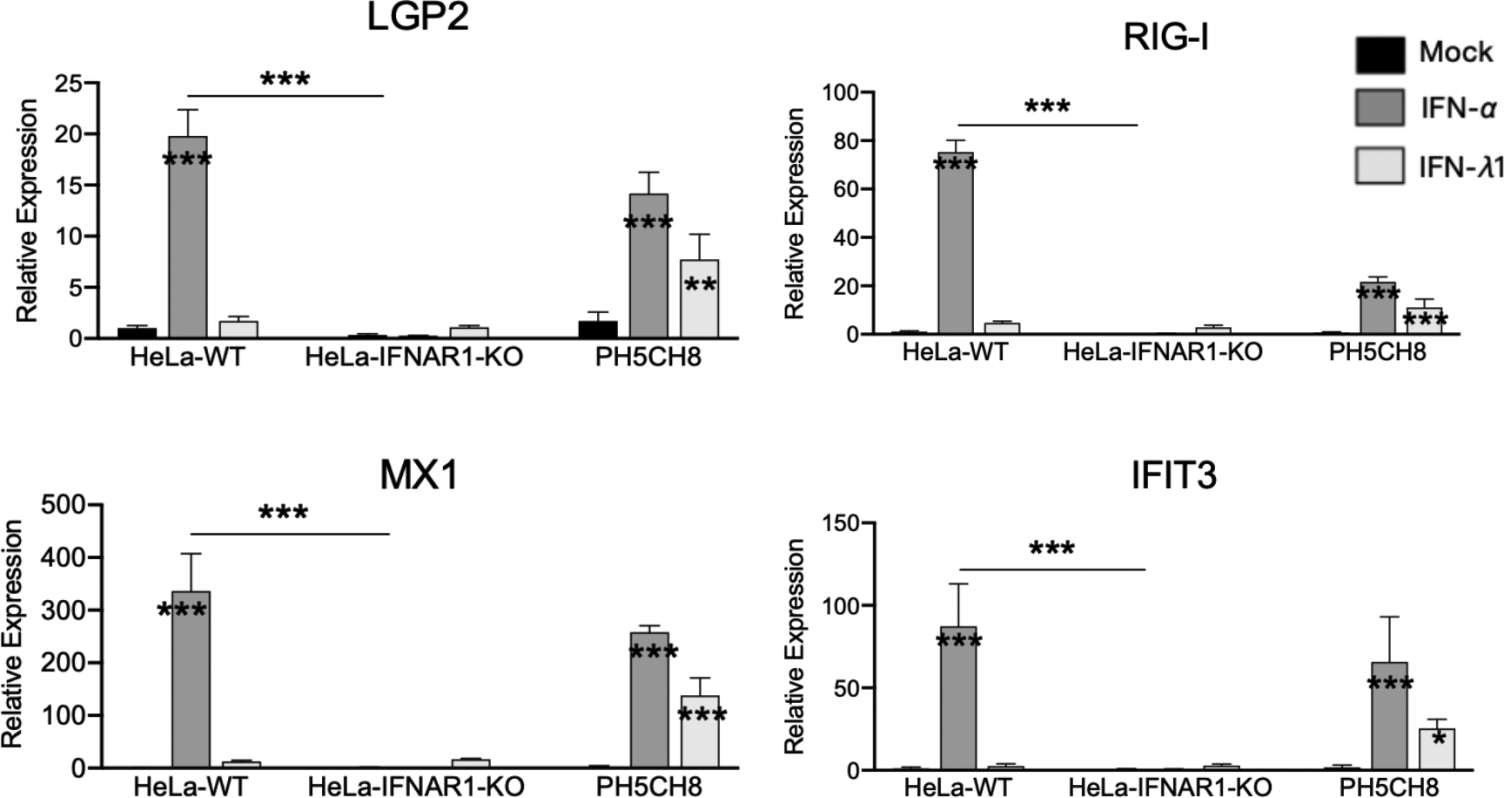
HeLa cells lack an ISG response to stimulation by type III IFN and HeLa-IFNAR1-KO cells are additionally defective for ISG induction by type I IFN. HeLa-WT, HeLa-IFNAR1-KO and PH5CH8 cells seeded at 1×10^5^ cells per well in 12-well plates were mock-treated or stimulated by recombinant human IFN-*α* (100 U ml^−1^) or IFN-*λ*1 (100 ng ml^−1^) for 8 h, followed by qRT-PCR quantification of transcripts for indicated ISGs with 28S rRNA used as the internal control for normalization. Data are from two independent experiments. Error bars represent sd. Asterisks inside bars represent the significance of the differences in gene expression between that treatment and that cell type’s mock treatment. Asterisks on the lines between bars represent the significance of the differences in gene expression between HeLa-WT and HeLa-IFNAR1-KO cells receiving that treatment. *P<0.05, **P<0.01, ***P<0.001.

At the outset of this study, we conducted experiments to validate the phenotypes of HeLa-WT and HeLa-IFNAR1-KO cells. Quantitative reverse transcription-PCR (qRT-PCR) that enables sensitive detection of the expression of ISGs was carried out to gauge the cellular responses to IFN-*λ*1, a type III IFN, and to IFN-*α*, a type I IFN. As a positive control, we included non-neoplastic hepatocyte PH5CH8 for side-by-side comparison. The relative expression of transcripts for LGP2 and three other antiviral ISGs (RIG-I, MX1 and IFIT3) was determined using gene-specific primers and normalized to that of 28S housekeeping control. As shown in Fig. 1, HeLa-WT cells exhibited no induction of any of the four ISGs – LGP2, RIG-I, MX1 or IFIT3 – following stimulation by IFN-*λ*1, although they responded robustly to IFN-*α* stimulation. By contrast, PH5CH8 exhibited considerable induction of all four ISGs following stimulation by either IFN type. As expected, HeLa-IFNAR1-KO cells lacked an ISG response to either IFN-*α* or IFN-*λ*1.

**Fig. 2.**
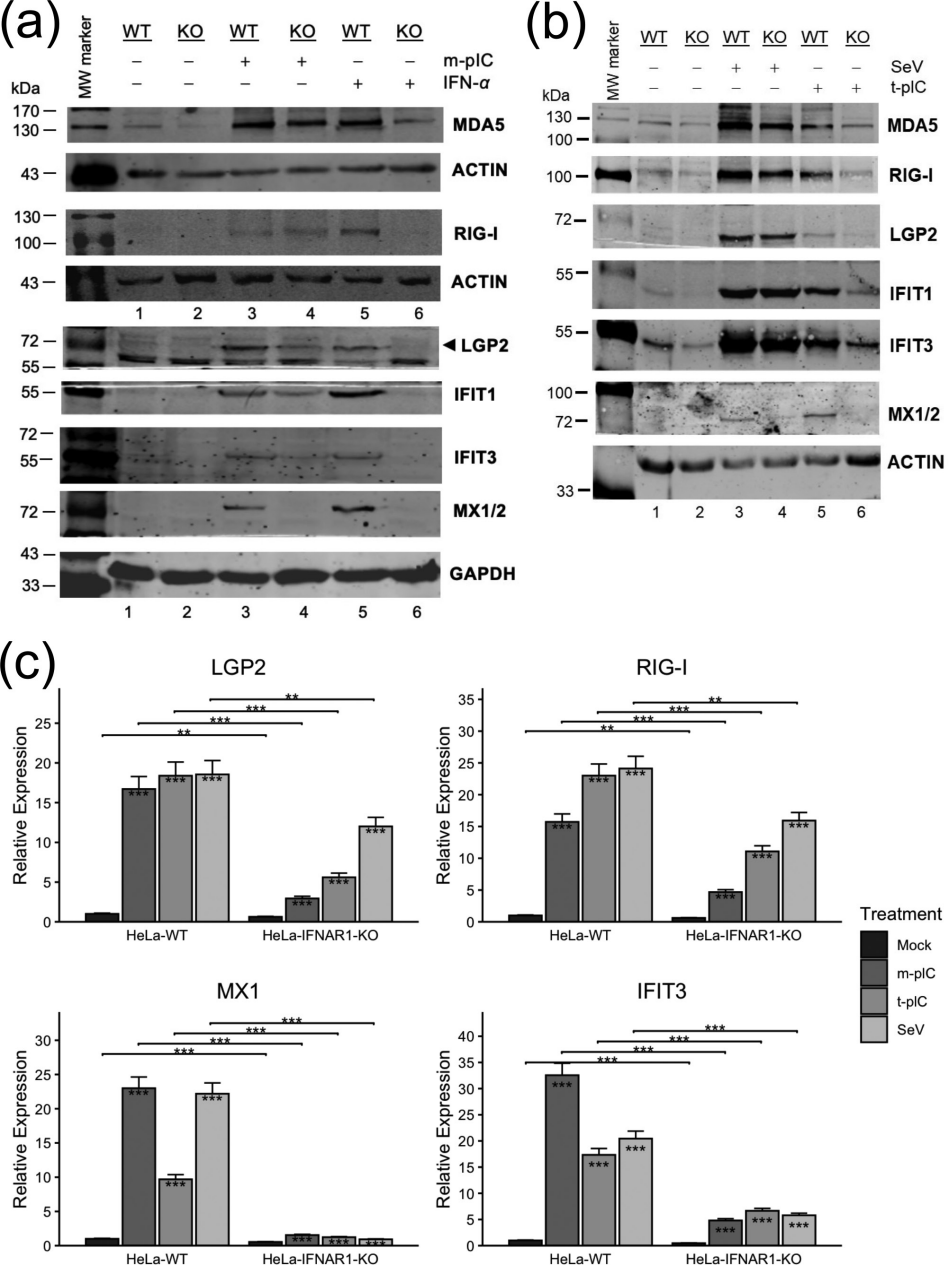
Induction of LGP2 expression via the TLR3 and RLR pathways in HeLa cells with or without IFNAR1 knockout. HeLa-WT and HeLa IFNAR1-KO cells were mock-stimulated or stimulated by poly(I:C) added directly to the culture medium (m-pIC) at a final concentration of 20 µg ml^−1^, recombinant IFN-*α* at a final concentration of 100 U ml^−1^, poly(I:C) complexed with Lipofectamine at 1 : 1 ratio (t-pIC) at 2 µg/well (35 mm dish size) or SeV at 200 HAU/ml. Sixteen hours later, cells were lysed for immunoblot analysis of the expression of indicated target proteins and a loading control (GAPDH or ACTIN) (a, b), or for total RNA extraction and cDNA synthesis, followed by qRT-PCR analysis of the abundance of indicated target mRNAs (c) (normalized to that of 28S). Fold changes for each gene were calculated relative to HeLa-WT mock group. The immunoblotting and qRT-PCR data presented are both representative of two independent experiments. Asterisks inside bars represent the significance of the differences in gene expression between that treatment and that cell type’s mock treatment. Asterisks on the lines between bars represent the significance of the differences in gene expression between HeLa-WT cells and HeLa-IFNAR1-KO cells receiving that treatment. *P<0.05, **P<0.01, ***P<0.001. Error bars represent ±sd.

As a starting point to investigate the regulation of LGP2 expression, we compared HeLa-WT and HeLa IFNAR1-KO cells for their responses to various stimuli mimicking viral infections. Initially, we tested extracellular poly(I:C), a dsRNA surrogate and ligand for TLR3 when administered directly to culture medium (m-pIC) [32], and recombinant IFN-*α*, respectively, and used mock-stimulated counterparts as controls. After 16 h, we performed immunoblotting to analyse the expression of three PRRs – RIG-I, MDA5 and LGP2, as well as several representative ISGs – IFIT1, IFIT3 and MX1/2 (Fig. 2a; quantification data presented in Fig. S1, available in the online Supplementary Material). While IFN-*α* stimulation substantially induced the expression of all above-mentioned antiviral proteins in HeLa-WT cells, albeit to varying degrees (compare lanes 5 vs 1), it failed to induce any of these in HeLa IFNAR-KO cells (compare lanes 6 vs 2), again validating the IFN signalling defect of the KO cells. As to the response to m-pIC stimulation, we observed that MX1/2 protein was robustly induced in HeLa-WT cells (compare lanes 3 vs 1) but completely undetectable in HeLa IFNAR1-KO cells (compare lanes 4 vs 2). This lack of expression in KO cells results from the fact that MX1/2 induction strictly depends on IFN produced by the stimulated cells, and transcription of the MX genes is not directly stimulated by activated IRF3 [21,22]. Since the KO cells lack IFNAR1 expression (and HeLa cells inherently lack IFNLR1 expression), the produced IFNs can no longer elicit the autocrine/paracrine signalling pathway that is essential for MX1/2 expression. In contrast, we observed that the expression of LGP2 protein was still substantially upregulated by m-pIC in HeLa IFNAR1-KO cells (compare lanes 4 vs 2) although to a lesser extent than in HeLa-WT cells (lane 3), indicating that LGP2 can be directly induced via the TLR3 pathway without an absolute requirement for prior IFN autocrine/paracrine signalling. This LGP2 induction pattern resembled that of MDA5, RIG-I, IFIT1 and IFIT3 (Fig. 2a; quantification data presented in Fig. S1), all of which can be upregulated directly by activated IRF3 [18,19, 23]. The reduced induction of LGP2 and the four ISGs of IRF3 target in HeLa IFNAR1-KO as compared with HeLa-WT cells can be explained by the fact that in the KO cells, the second wave of gene induction – IFN autocrine/paracrine signalling-dependent amplification via the Jak-STAT pathway – is cut off.

To determine whether the IFN-independent viral induction of LGP2 also operates via pathways other than TLR3, we used SeV infection and cytoplasmic delivery of poly(I:C) by Lipofectamine transfection (t-pIC), respectively, to engage the cytosolic RLRs, RIG-I and MDA5, and assessed the downstream antiviral response (Fig. 2b; quantification data presented in Fig. S2). We obtained similar results to those observed with TLR3 stimulation − in HeLa IFNAR1-KO cells, LGP2 was still upregulated substantially following SeV challenge or t-pIC stimulation as were MDA5, RIG-I, IFIT1 and IFIT3, albeit at reduced levels than in HeLa-WT cells, while MX1/2 expression remained undetectable in IFNAR1-KO cells following RLR engagement as opposed to its readily detectable induction in WT cells. Taken together, these data demonstrate that as in the case with ISGs of direct IRF3 target, LGP2 does not strictly depend on prior IFN production and signalling for its upregulation by viral stimuli via each of the dsRNA-sensing pathways.

Next, we performed qRT-PCR to assess the abundance of transcripts for LGP2 and several other antiviral genes (RIG-I, IFIT3 and MX1) (Fig. 2c). The results showed that the induction trends for all these genes by m-pIC, t-pIC and SeV, respectively, were consistent with the immunoblotting data (Fig. 2a, b), suggesting that the uptick in their expression following activation of the TLR3 and RLRs pathways is mainly governed at the mRNA level. Notably, in the absence of IFN signalling, induction of MX1 mRNA was abrogated, conforming to the established paradigm that the upregulation of MX genes in viral infection requires IFN paracrine/autocrine signalling. In contrast, when compared to HeLa-WT cells, the upregulation of LGP2, RIG-I and IFIT3 mRNAs in HeLa IFNAR1-KO cells was only reduced (to varying extent depending on gene and stimulus), with considerable induction that remained. Thus, unlike the MX proteins, the expression of these antiviral genes, including LGP2, can be activated through IFN-independent mechanisms, although IFN autocrine/paracrine signalling enables their maximal induction.

To understand the kinetics of LGP2 induction, we conducted qRT-PCR to determine the expression of LGP2 and SeV phosphoprotein gene, in comparison with that of RIG-I, MX1 and IFIT3, in HeLa-WT and HeLa-IFNAR1-KO cells, mock-infected and infected by SeV at 6 and 12 h post-infection (h.p.i.), respectively. The results showed that the upregulation of all four ISGs in HeLa-WT cells and that of LGP2, RIG-I and IFIT3 in HeLa-IFNAR1-KO cells (no induction of MX1 in the KO cells) followed similar kinetics and correlated with the extent of viral replication (i.e. the levels of viral PAMP, in this case SeV RNA): there was little induction at 6 h when SeV replication was very low while robust induction at 12 h when abundant viral RNA was present (Fig. S3).

### Considerable induction of LGP2 by cytosolic dsRNA or virus in non-neoplastic hepatocyte PH5CH8 remains despite efficient chemical inhibition of JAK-STAT signalling

To further corroborate that virus-inducible LGP2 expression occurs in the absence of IFN autocrine/paracrine signalling, we took a chemical inhibition approach. We tested ruxolitinib, a potent, selective inhibitor of JAK1/2 kinase activity [42] that blocks the induction of ISGs via the Jak-STAT pathway by all three IFN types as well as by a variety of inflammatory cytokines, including IL-6, IL-12, IL-23 and GM-CSF, among others. For this experiment, we selected PH5CH8 cells, a non-neoplastic human hepatocyte line, which harbours intact antiviral innate immune pathways [24,32]. This cell model enabled us to study the response of non-malignant cells and to validate our initial findings with HeLa-derived cells, which are of human cervical epithelial carcinoma origin.

**Fig. 3.**
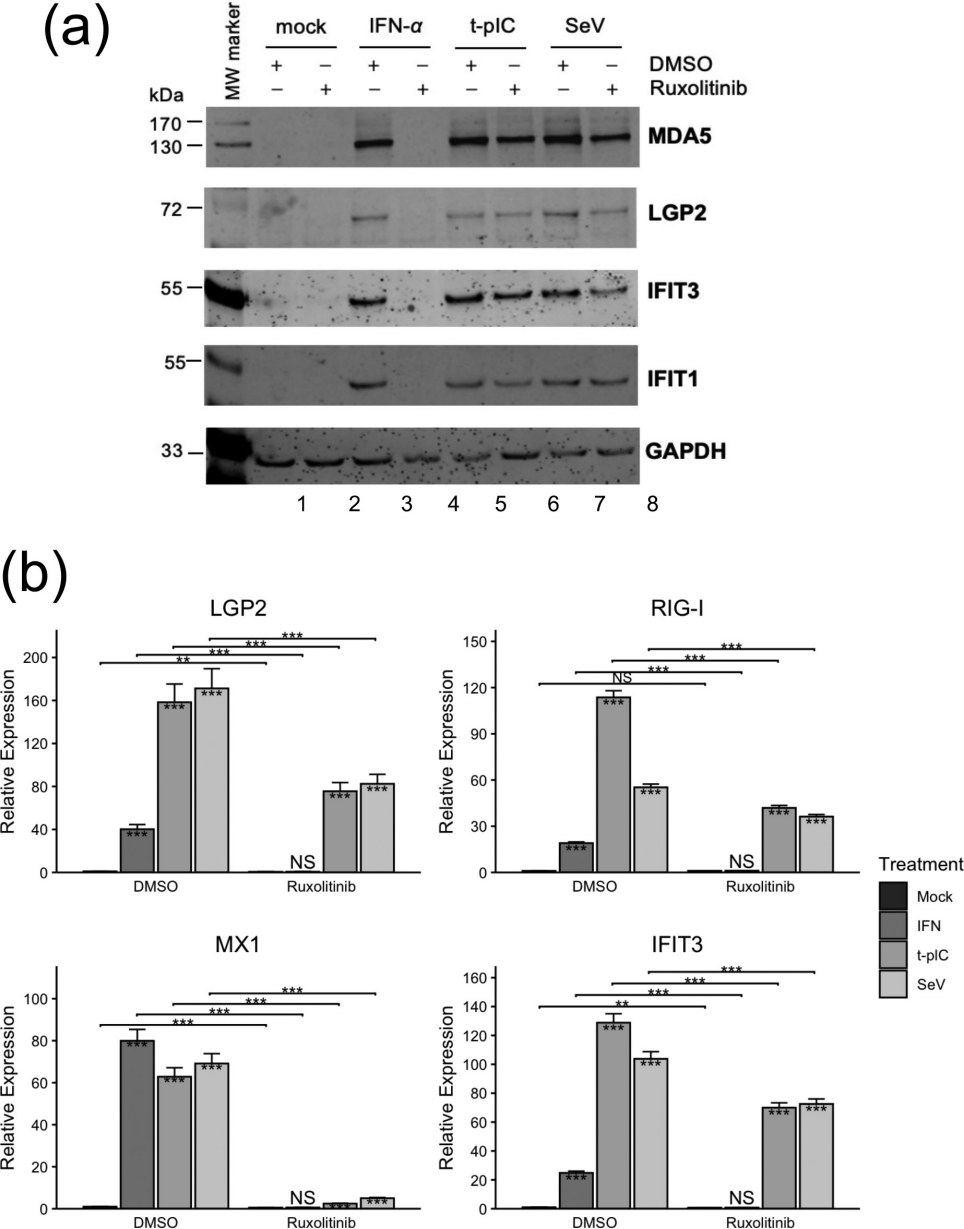
Induction of LGP2 expression by dsRNA or virus in non-neoplastic hepatocyte PH5CH8 cells in the presence or absence of a pan-JAK inhibitor, ruxolitinib. PH5CH8 cells seeded on 6-well plates at 4×10^5^/well overnight were pretreated for 2 h in medium containing 10 µM ruxolitinib or DMSO (solvent control, at a final concentration of 0.1%). Subsequently, cells were mock-stimulated, incubated with 100 U ml^−1^ recombinant human IFN-α, transfected with poly(I:C) (at 0.5 µg per well) or infected with 200 HAU/ml SeV, in the presence of DMSO or ruxolitinib, respectively. At 16 h post-stimulation, cells were lysed for immunoblotting (**a**) or RNA extraction and qRT-PCR (**b**) analyses of the expression of LGP2 and other indicated targets. The immunoblotting and qRT-PCR data are representative of two and three independent experiments, respectively. Statistical analyses were conducted as those in Fig. 1(c). *P<0.05, **P<0.01, ***P<0.001. ns, no significance.

We pretreated PH5CH8 cells with ruxolitinib for 2 h, followed by mock stimulation or stimulation by recombinant IFN-*α*, pIC transfection or SeV infection in the presence of ruxolitinib. For negative control groups, we used DMSO (solvent) in lieu of ruxolitinib. We then assessed the changes in expression of LGP2 and other representative ISGs by immunoblotting (Fig. 3a, quantification data presented in Fig. S4). The data showed that compared to the mock-stimulation group, IFN-*α* robustly induced the expression of LGP2, MDA5, IFIT1 and IFIT3 proteins in the presence of DMSO (compare lanes 3 vs 1). However, in the presence of Ruxolitinib, none of the four antiviral proteins was detectable in IFN-α-stimulated cells (compare lanes 4 vs 2). This result confirmed the effectiveness of ruxolitinib treatment in blocking Jak-STAT signalling, the effector mechanism of ISG induction by IFNs and a subset of inflammatory cytokines. When we examined the ISG induction by pIC transfection or SeV infection via the RLR-dependent pathway, we found that all four antiviral proteins including LGP2 were strongly induced by either stimulus, with their expression levels only moderately reduced in the presence of ruxolitinib compared with DMSO control (compare lanes 6 vs 5, and lanes 8 vs 7, respectively). We also performed qRT-PCR to validate these results at the RNA level. As shown in Fig. 3(b), while ruxolitinib completely ablated the upregulation of LGP2, RIG-I, IFIT3 and MX1 mRNAs by IFN-*α*, it reduced the expression of LGP2 mRNA in cells stimulated by SeV or t-pIC by only ~50%. Likewise, ruxolitinib only partially curtailed the induction of RIG-I and IFIT3 mRNAs by either stimulus (by ~30–60% depending on gene and stimulus). In stark contrast, ruxolitinib almost eliminated MX1 mRNA induction by SeV or t-pIC, reducing it by ~92–96%. In aggregate, these data confirm that a considerable fraction of LGP2 expression during viral infection is independent of IFN and cytokine autocrine/paracrine signalling via the Jak kinases in non-malignant cells.

### Overexpression of IRF3-5D or RELA upregulates LGP2 protein and mRNA levels in both HeLa-WT and HeLa-IFNAR1-KO cells

**Fig. 4.**
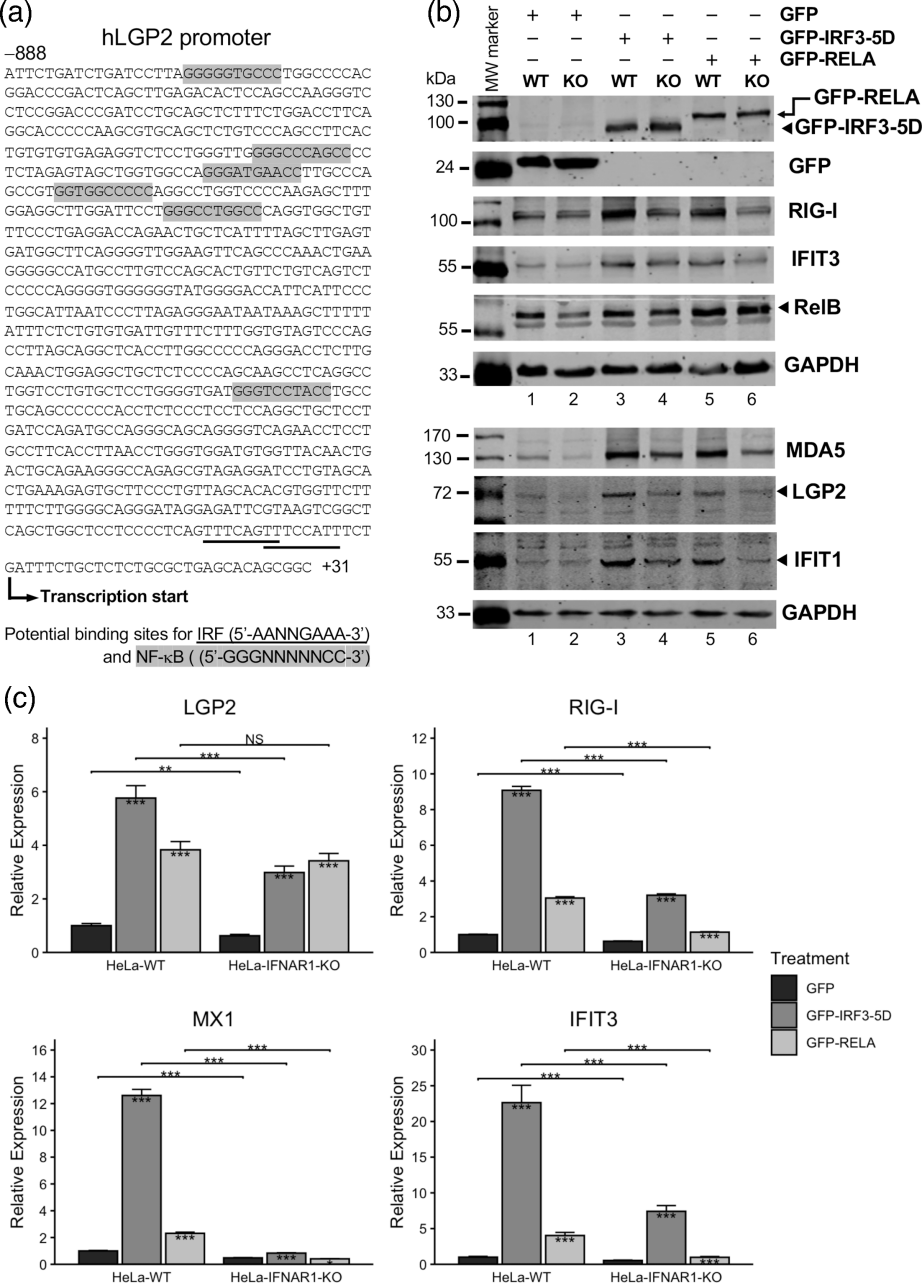
Induction of LGP2 expression by overexpression of a constitutively active, phospho-mimetic IRF3 mutant (IRF3-5D), or to a lesser extent, the RELA (p65) subunit of NFκB in HeLa cells with or without IFNAR1 knockout. (**a**) Annotated sequence of the human LGP2 promoter (−888 ~ +31). The putative ISRE/IRF-E (in reverse complement orientation) and NFκB binding sites are underlined and grey-shaded, respectively. (**b, c**) HeLa WT and HeLa IFNAR1-KO cells were transfected with GFP empty vector, GFP-IRF3-5D and GFP-RELA, respectively at 2 µg/well (b, on 6-well plates) or 1 µg/well (c, on 12-well plates), for 48 h (**b**) or 24 h (**c**). Cells were then lysed for immunoblotting (**b**) or RNA extraction and qRT-PCR (**c**) analyses of the expression of LGP2 and other indicated targets. Note that in (**b**), a mouse mAb anti-GFP was used to detect the expression of ectopically transfected GFP, GFP-IRF3-5D and GFP-RELA, while a rabbit pAb anti-RELB was used to probe the expression levels of endogenous RELB protein. The immunoblotting and qRT-PCR data are both representative of three independent experiments. *P<0.05, **P<0.01, ***P<0.001.

IRF3 and NFκB represent the two key transcription factors regulating the expression of type I and type III IFNs and some antiviral ISGs. Since we observed that LGP2 mRNA could be upregulated by dsRNA or virus when IFN signalling via its receptors and downstream Jak-STAT signalling is absent, we considered the possibility that activated IRF3 and/or NFκB could directly regulate the transcription of LGP2. Indeed, inspection of the human LGP2 promoter sequence up to ~–890 nt from the transcription start site found two overlapping IRF recognition consensus sequences (5′-AANNGAAA-3′) [43] (Fig. 4a, underlined) and several potential NFκB binding sites (Fig. 4a, grey-shaded) conforming to the consensus sequence (5′-GGGNNNNNCC-3′) [44]. To determine the effects of IRF3 and NFκB on LGP2 expression, we transfected HeLa-WT and HeLa IFNAR1-KO cells with a plasmid vector encoding GFP alone (as a negative control), GFP-IRF3-5D [34] and GFP-p65 (RELA) [35], respectively. The 5D mutant of IRF3 mimics the C-terminal phosphorylated (activated) form of IRF3, which is capable of promoting the expression of type I and type III IFNs and other antiviral genes when ectopically expressed [34,45]. Regarding p65 (a.k.a., RELA), it is a major subunit of the NFκB family in terms of the transcriptional activity and, when overexpressed, drives activation of the NFκB signalling pathway and expression of downstream target genes [35]. At 48 h post-transfection, we assessed the expression of LGP2 and several other ISGs as well as RELB, a specific NFκB target. Immunoblotting (Fig. 4b, quantification data presented in Fig. S5) using an anti-GFP monoclonal antibody confirmed successful expression of each of the three plasmid constructs. The immunoblot analysis revealed that compared to the GFP control group, expression of GFP-IRF3-5D led to heightened expression of LGP2, MDA5, RIG-I, IFIT3 and IFIT1 in both HeLa-WT and HeLa IFNAR1-KO cells, albeit to a lesser extent in the latter. This indicates that activated IRF3 drives LGP2 expression, as it does other IRF3-target genes and that this effect does not rely on IFN autocrine or paracrine signalling. Regarding GFP-RELA, we observed that its overexpression resulted in the upregulation of RELB in both HeLa-WT and HeLa IFNAR1-KO cells, when compared to the GFP control group. This confirms the function of GFP-RELA in activating the expression of NFκB-dependent genes. Overall, enforced expression of GFP-RELA also upregulated LGP2 and other ISGs in HeLa-WT cells, but its effect was notably weaker than that of GFP-IRF3-5D. Interestingly, while deletion of IFNAR1 nullified the impact of GFP-RELA on increasing the expression of RIG-I, IFIT3 and IFIT1, it had a minor effect on that of MDA5 and did not impact much the protein levels of LGP2 (Fig. 4b). The observation that GFP-RELA alone drove ISG upregulation in HeLa-WT cells, albeit moderately, was a bit unexpected, given the current consensus that NFκB activation per se is not sufficient for virus-induced type I IFN transcription. While an overexpression effect cannot be ruled out, it is of note that RELA maintains constitutive expression of IFN-*β* and a subset of ISGs in the absence of viral infection [46]. Conceivably, this function is lost when IFN autocrine signalling is blocked, and RELA may contribute to direct transcriptional control of specific ISGs such as MDA5 and LGP2. To what extent this is the case will require future investigation. Altogether, these data show that activated IRF3, and to a lesser extent NFκB, can directly upregulate the expression of LGP2 and that these effects do not require intact IFN autocrine/paracrine signalling.

We further confirmed these results by qRT-PCR. As shown in Fig. 4(c), ectopic expression of GFP-IRF3-5D, and to a lesser extent GFP-RELA, increased the mRNA levels of LGP2, RIG-I, IFIT3 and MX1 in HeLa-WT cells compared to the GFP vector group. The loss of IFNAR1 (i.e. IFN autocrine/paracrine signalling) rendered GFP-IRF3-5D less effective in driving up the expression of LGP2, RIG-I and IFIT3 and negated its effect on MX1, a strictly IFN-dependent gene. When it came to the case of GFP-RELA, IFNAR1 deletion severely compromised, if not eliminated, the moderate effects on induction of RIG-I and IFIT3 and completely abrogated the effect on MX1 induction. In contrast, the upregulation of LGP2 by GFP-RELA was not found to be affected by IFNAR1-KO. Thus, apart from corroborating the Fig. 4(b) immunoblotting data, these RNA data confirm that the NFκB activity driven by RELA moderately increases LGP2 expression, an effect not hinging on IFN induction and signalling. The opposite should be said when it comes to the effects of RELA on RIG-I, IFIT3 and MX1 expression, which our data suggest operate indirectly via IFN’s autocrine/paracrine action.

### Knockdown of IRF3 or chemical inhibition of NFκB undermines virus-induced LGP2 expression in HeLa-WT and HeLa-IFNAR1-KO cells

**Fig. 5.**
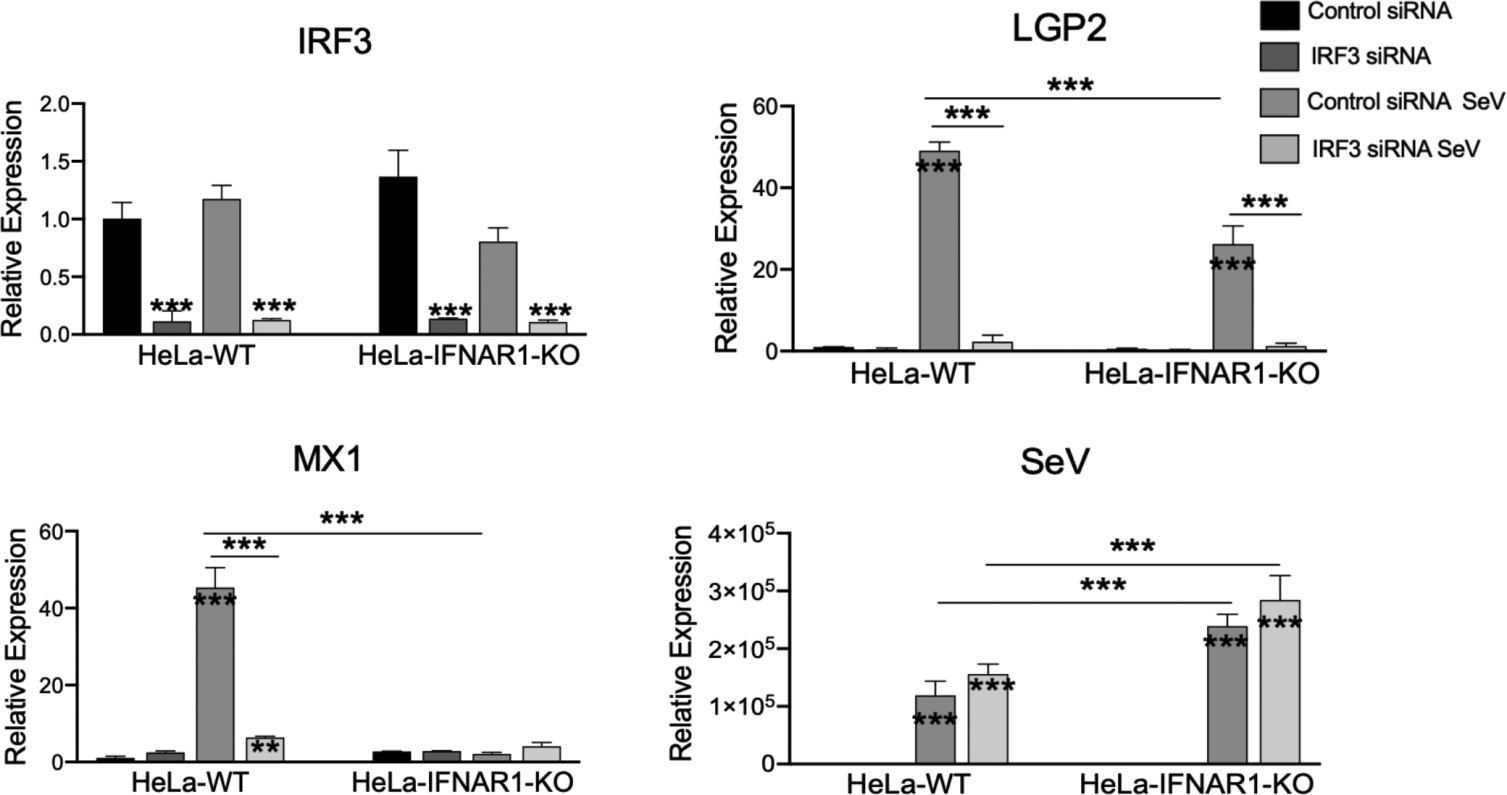
RNAi depletion of IRF3 diminishes SeV-induced LGP2 expression as determined by qRT-PCR. HeLa-WT and HeLa-IFNAR1-KO cells seeded at 0.5×10⁵ cells per well in 12-well plates were transfected with either a non-targeting, negative control siRNA or siRNA against IRF3 (2 µl of 40 µM siRNA stock per well). After 48 h, cells were mock infected or infected by SeV at 200 HAU/ml for 16 h. Total RNA was then extracted and reverse-transcribed into cDNA, followed by qRT-PCR analysis of IRF3, LGP2, MX1 and SeV mRNA levels (normalized to 28S internal control). Data are from two independent experiments. Error bars represent sd. Asterisks inside/on top of bars represent the significance of the differences in gene expression between that treatment and that cell type’s mock treatment. Asterisks on the lines between bars represent the significance of the differences in gene expression between the groups as indicated. *P<0.05, **P<0.01, ***P<0.001.

**Fig. 6.**
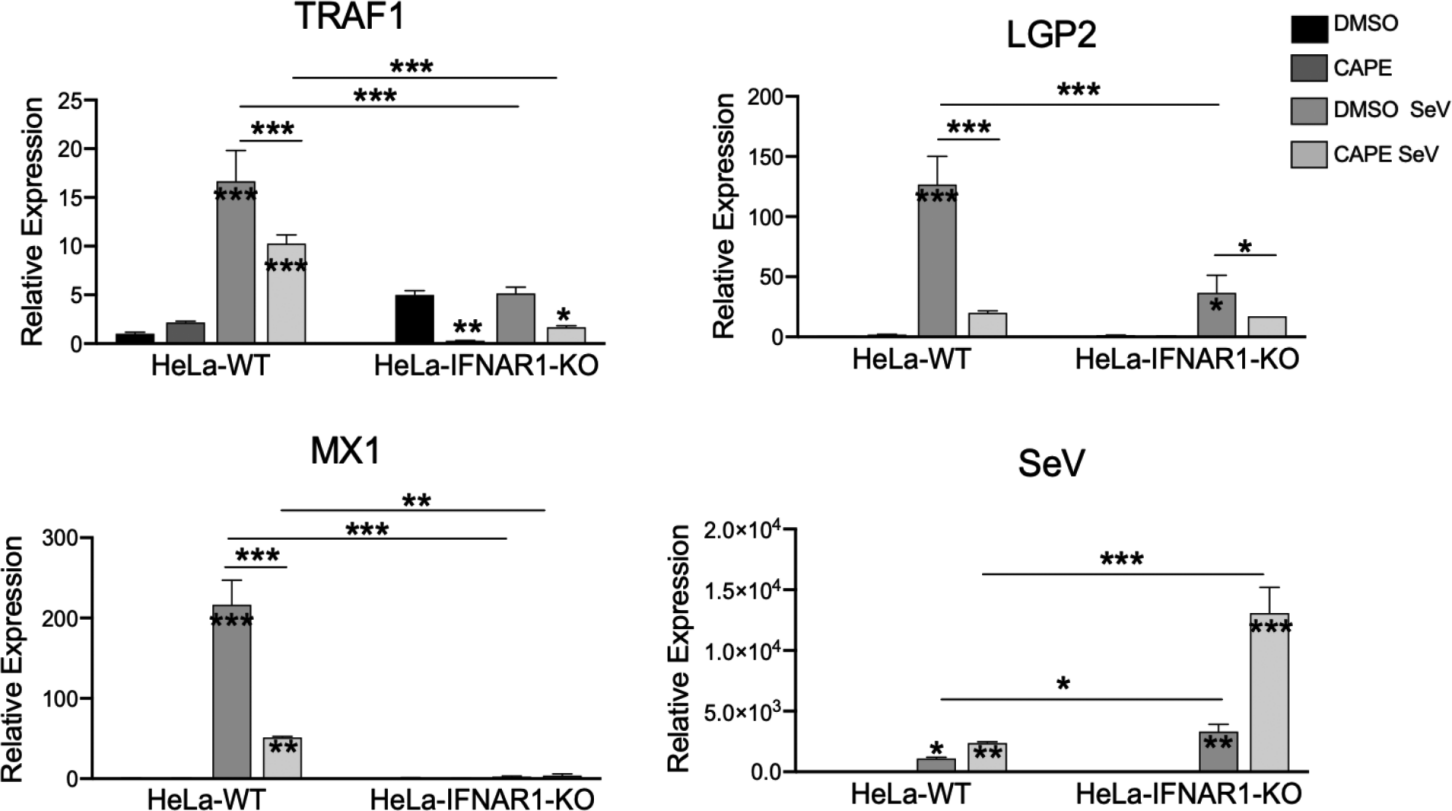
Chemical inhibition of NFκB activation curtails SeV-induced LGP2 expression as determined by qRT-PCR. HeLa-WT and HeLa-IFNAR1-KO cells seeded at 0.8×10^5^ cells per well in 12-well plates were pretreated for 2 h with either DMSO (solvent control) or CAPE (20 µM), a selective NFκB inhibitor. Cells were then mock-infected or infected by SeV at 200 HAU/ml for 16 h in the presence of DMSO or CAPE. Subsequently, total RNA was isolated and reverse-transcribed into cDNA, followed by qRT-PCR analysis of TRAF1, LGP2, MX1 and SeV mRNA levels (normalized to 28S internal control). Data are from two independent experiments. Error bars represent sd. Asterisks inside/on top of bars represent the significance of the differences in gene expression between that treatment and that cell type’s mock treatment. Asterisks on the lines between bars represent the significance of the differences in gene expression between the groups as indicated. *P<0.05, **P<0.01, ***P<0.001.

To verify that IRF3 and NFκB each contribute to virus-induced LGP2 expression in a physiological setting, we determined the effects of IRF3 knockdown and chemical inhibition of NFκB activation, respectively, in HeLa-WT and HeLa-IFNAR1-KO cells on ISG expression in response to SeV infection by qRT-PCR. As shown in Fig. 5, transient transfection of an IRF3-specific siRNA diminished IRF3 mRNA expression, as compared with that of a non-targeting, negative control siRNA. Concomitantly, IRF3 depletion abrogated SeV-induced expression of LGP2 in both HeLa-WT and HeLa-IFNAR1-KO cells, as it did viral induction of MX1 in HeLa-WT cells (which is dependent on IFN autocrine/paracrine signalling as SeV infection did not lead to MX1 expression in HeLa-IFNAR1-KO cells despite higher, more robust viral replication in the latter). The role of NFκB was investigated using a specific chemical inhibitor, CAPE [47]. As illustrated in Fig. 6, we observed that CAPE significantly reduced SeV-induced upregulation of LGP2 mRNA in both HeLa-WT and HeLa-IFNAR-KO cells and that of MX1 mRNA in HeLa-WT cells, as compared to DMSO solvent control. As a positive control, the expression of TRAF1, a known NFκB target gene, was significantly reduced by CAPE. Together, these experiments corroborated the critical roles of endogenous IRF3 and NFκB in virus-induced expression of LGP2.

### Ectopic expression of IRF3-5D or RELA drives the activation of human LGP2 promoter

**Fig. 7.**
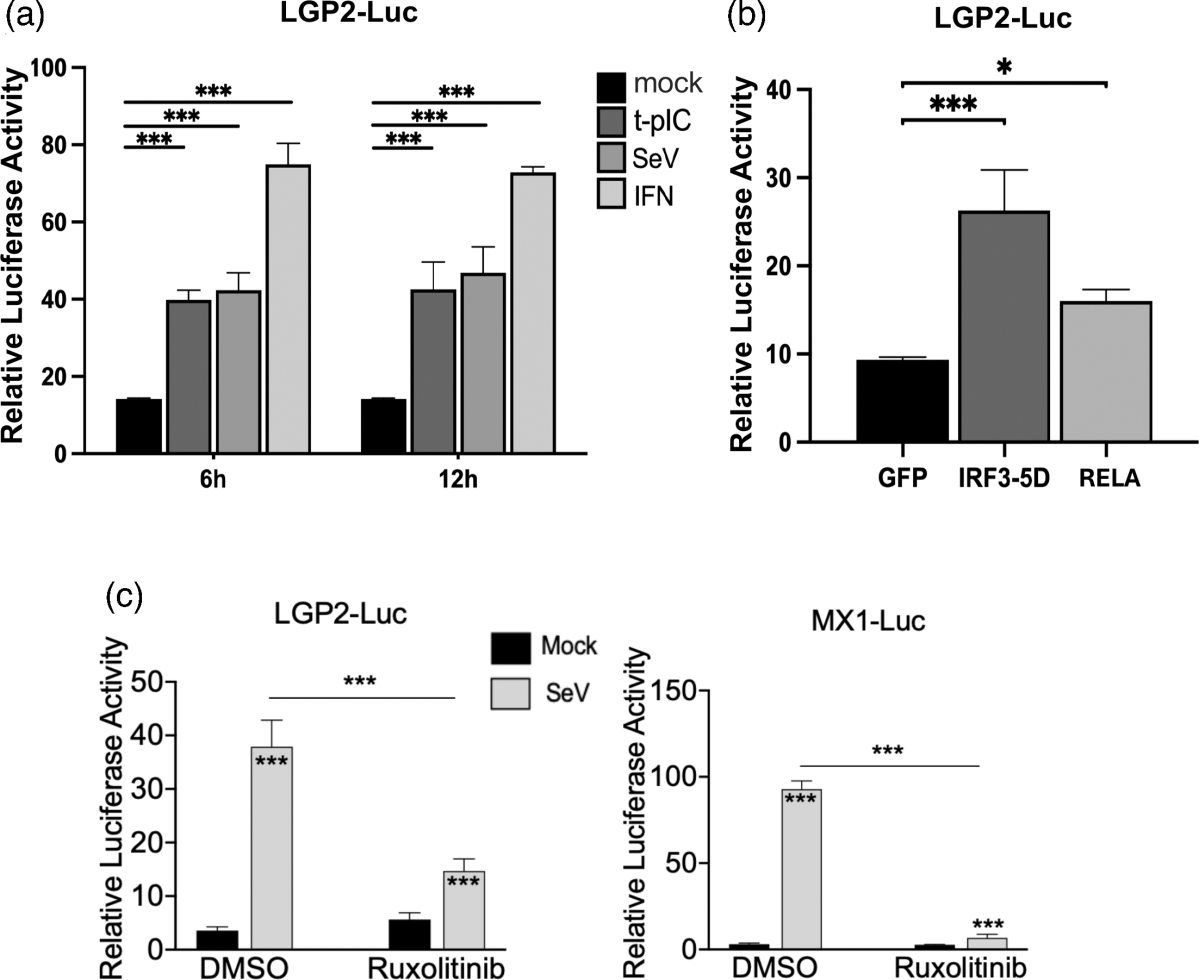
Upregulation of LGP2 expression at the promoter level by various stimuli. (**a**) HEK293A cells seeded at 4×10^4^ cells/well on 48-well plates were co-transfected with pGL3-hLGP2 (at 80 ng/well) and pRL-TK (Promega, as an internal control, at 20 ng/well). After 42 h, triplicate wells of cells were mock-treated, stimulated with recombinant IFN-*α* (at 100 U ml^−1^) or poly(I:C) complexed with Lipofectamine at 1 : 1 ratio (t-pIC, at 1 µg/well) or challenged by SeV at 200 HAU/ml. At 6 h and 12 h post-stimulation, cells were lysed in passive lysis buffer (50 µl/well) and subjected to dual-luciferase reporter assay (Promega). (**b**) Triplicate wells of HEK293 cells grown on 48-well plates were co-transfected with pGL3-hLGP2 (80 ng/well) and pRL-TK (20 ng/well), along with 100 ng/well of GFP empty vector (as a negative control), or GFP-IRF3-5D or GFP-RELA, respectively. At 24 h post-transfection, cells were lysed for dual luciferase assay as in (**a**). (**c**) PH5CH8 cells seeded in 48-well plates at 2×10^4^ cells/well were co-transfected with pRL-TK (30 ng/well) and pGL3-hLGP2 (120 ng/well, left) or pGL4-MX1 (120 ng/well, right). After 24 h, cells were divided into two groups: one pre-treated with DMSO (solvent control) and the other with ruxolitinib (10 µM) for 2 h. Subsequently, cells were mock-infected or infected with SeV (200 HAU/ml) in the presence of DMSO or ruxolitinib, respectively. At 16 h.p.i., cells were lysed for dual-luciferase assay as in (**a**). Data are expressed as relative luciferase activities (i.e. promoter activities) under each condition and are representative of two independent experiments (mean±sd). *P<0.05, **P<0.01, ***P<0.001. Asterisks inside or on top of bars represent the significance of the differences as compared with the respective mock group, while those on the lines between bars represent the significance of the differences between the groups as indicated.

To further understand the mechanism of LGP2 mRNA upregulation during viral infection, we sought to determine whether increased transcription of the gene is a contributing factor. To this end, we created a luciferase reporter plasmid in which a human LGP2 promoter DNA fragment spanning nt positions –888 to +31 (Fig. 4a) was placed upstream of the firefly luciferase reporter gene. This reporter construct, which we designated pGL3-hLGP2, would enable convenient and quantitative monitoring of transcriptional activity from the LGP2 promoter in transfected cells by measuring the enzymatic activity of luciferase reporter protein produced. To test the function of pGL3-hLGP2, we introduced the construct into HEK293A cells, which allow for higher transfection efficiency than HeLa cells, by Lipofectamine. A control plasmid, pRL-TK, that encodes the Renilla luciferase gene under control of the constitutive, thymidine kinase promoter, was co-transfected with pGL3-hLGP2 for normalization of any variation in transfection efficiency. Subsequently, transfected cells were mock-stimulated or stimulated by poly(I:C) transfection, SeV infection and recombinant IFN-*α*, respectively. Dual luciferase assay of the cell lysates showed significant increases in transcription from the LGP2 promoter under all three treatment conditions than mock controls, indicating that pGL3-hLGP2 harbours the necessary sequence elements of human LGP2 promoter critical for recapitulating its transcriptional regulation. Specifically, at 6 h post-stimulation, compared to the mock control group, the three conditions enhanced LGP2 promoter activity by 2.8-fold (t-pIC), 3.0-fold (SeV) and 5.3-fold (IFN-*α*), respectively (*P*<0.001). At 12 h post-stimulation, there was not much difference compared to the earlier time point measurements (3.5-fold, 3.3-fold and 5.1-fold, respectively) (Fig. 7a).

Having demonstrated that human LGP2 gene promoter is activated by cytosolic dsRNA (t-pIC), SeV or IFN-*α*, we next determined whether the IRF3-5D and RELA upregulations of LGP2 mRNA expression observed earlier (Fig. 4) stem from their abilities to activate the gene transcription at the promoter level. To achieve this, we co-transfected HEK293 cells with pGL3-hLGP2 and pRL-TK, along with either the GFP alone-encoding vector or a vector encoding GFP-IRF3-5D or GFP-RELA, and conducted dual-luciferase assay to gauge the transcriptional activities of human LGP2 promoter. As shown in Fig. 7(b), ectopic expression of either GFP-IRF3-5D or GFP-RELA led to significantly increased LGP2 promoter transcription compared to the GFP control group. Specifically, IRF3-5D enhanced the promoter activity by 3.1-fold (*P*<0.001), while RELA raised it by 1.7-fold (*P*<0.05) (Fig. 7b). The greater ability of IRF3-5D than RELA was consistent with our earlier data measuring endogenous LGP2 mRNA levels (Fig. 4c).

### Viral activation of human LGP2 promoter remains at substantial level in PH5CH8 cells when JAK-STAT signalling is strongly inhibited

Finally, to validate that human LGP2 promoter can be activated by virus infection in an IFN- and cytokine-independent manner in non-malignant cells, we performed dual luciferase reporter-based promoter activation assay in PH5CH8 cells, with and without pre-treatment of ruxolitinib, followed by mock-infection and SeV infection, respectively. PH5CH8 cells responded to SeV challenge more robustly than HEK293A or HEK293 cells, enabling a ~10.6 fold induction of the LGP2 promoter. This response was partially reduced (by ~70%) by ruxolitinib (Fig. 7c, left panel). In contrast, the SeV activation of human MX1 promoter exhibited a much greater dependence on JAK-STAT signalling – in PH5CH8 cells transfected with pGL4-MX1 (in which human MX1 promoter was placed upstream of firefly luciferase gene) [33] in lieu of pGL3-hLGP2, ruxolitinib almost eliminated the increase in SeV-induced reporter activity (Fig. 7c, right panel).

## Discussion

We have revealed in this study new aspects of regulation of LGP2/DHX58, the third RLR member, in innate antiviral immune responses, by uncovering a cytokine-independent mode that operates in human cells to upregulate this viral RNA sensor during viral infection. Importantly, we have shown this mechanism to function in both cancer and non-malignant cells of distinct tissue origins and via multiple PRR pathways. Mechanistically, we demonstrate that IRF3 and NFκB each contribute to orchestrating the transcription of LGP2 mRNA from its promoter, independent of IFN autocrine/paracrine signalling. While LGP2 is well documented in the literature as an ISG, our data demonstrate that a significant fraction of LGP2 induction during viral infection takes place in the absence of IFN autocrine/paracrine signalling, with the latter contributing to the gene’s maximal induction. This revelation underscores a host evolutionary advantage in tackling viral infections. As many viruses have acquired the ability to block the induction and/or signalling of IFNs, it is plausible that the IFN- and inflammatory cytokine-independent augmentation of LGP2 transcription by IRF3 and/or NFκB may facilitate an uninterrupted supply of this key player, ensuring that timely and effective innate antiviral responses are mounted. To what extent this regulatory mechanism may negatively impact RIG-I-dependent host responses to some viral infections will require further investigation. Future studies are warranted to pinpoint the exact genetic elements in LGP2 promoter through which IRF3 and NFκB exert their actions and determine whether other IRFs may also play a part, to aid a fuller understanding of the regulatory mechanisms of LGP2 expression.

## Supplementary material

10.1099/jgv.0.002173 Uncited Supplementary Material 1.

## Abbreviations

CAPE: caffeic acid phenethyl ester
CARD: caspase activation and recruitment domain
h.p.i.: hours post-infection
IRF3: IFN-regulatory factor-3
ISG: IFN-stimulated gene
LGP2: laboratory of genetics and physiology 2
MAVS: mitochondrial antiviral signalling
PAMP: pathogen-associated molecular pattern
PRR: pattern recognition receptor
qRT-PCR: quantitative reverse transcription-PCR
RLR: RIG-I-like receptor
SeV: Sendai virus
TLR3: Toll-like receptor 3
