# Supplementary material for: Cytokine-independent induction of LGP2/DHX58 in viral infection
Source: J Gen Virol. 2025 Oct 31;106(10):002173. doi: 10.1099/jgv.0.002173 (PMC12578130; doi:10.1099/jgv.0.002173)
Supplement: Uncited Supplementary Material 1. [file jgv-106-02173-s001.pdf]

Supplementary Figures

Cytokine-independent Induction of LGP2/DHX58 in Viral Infection

Yaxin Liu, Xiaohan Tong, Ruixue Wang, Z. Galvin Li, Zichen Xie, Dang Wang, Weikuan Gu, and Kui Li

This file contains Supplementary Figures S1 to S5.

Figure S1

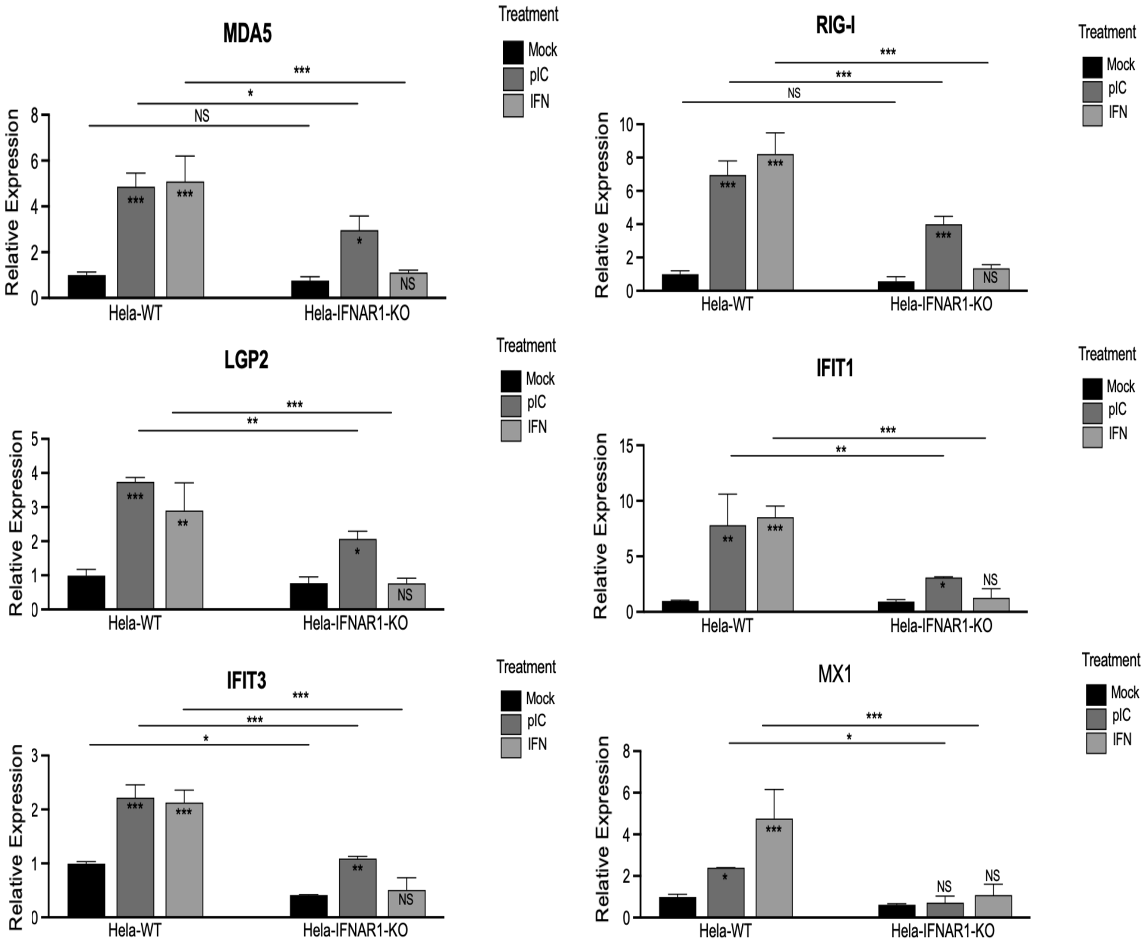

Figure S1. Quantification of Figure 2A immunoblotting data by densitometry analysis. Data are from two independent immunoblotting experiments (normalized to a housekeeping loading control) and analyzed by Image Studio Lite (LI-COR Biosciences). Error bars represent SD. Asterisks inside bars represent the significance of the differences in expression between that treatment and that cell type's mock treatment. Asterisks on the lines between bars represent the significance of the differences in expression between the indicated groups. *$p < 0.05$, **$p < 0.01$, ***$p < 0.001$. NS, not significant.

Figure S2

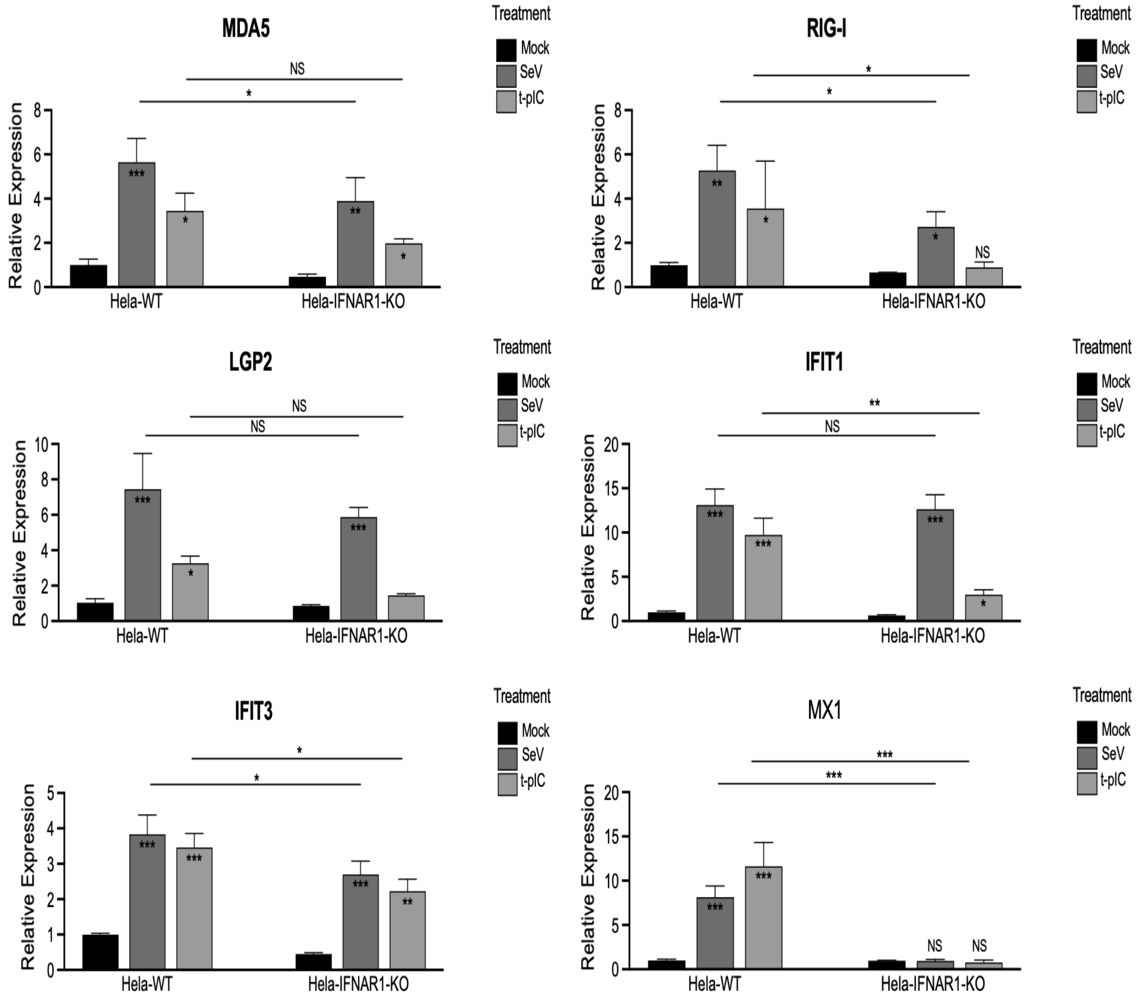

Figure S2. Quantification of Figure 2B immunoblotting data by densitometry analysis. Data are from two independent immunoblotting experiments (normalized to a housekeeping loading control) and analyzed by Image Studio Lite (LI-COR Biosciences). Error bars represent SD. Asterisks inside bars represent the significance of the differences in expression between that treatment and that cell type's mock treatment. Asterisks on the lines between bars represent the significance of the differences in expression between the indicated groups. *$p < 0.05$, **$p < 0.01$, ***$p < 0.001$. NS, not significant.

Figure S3

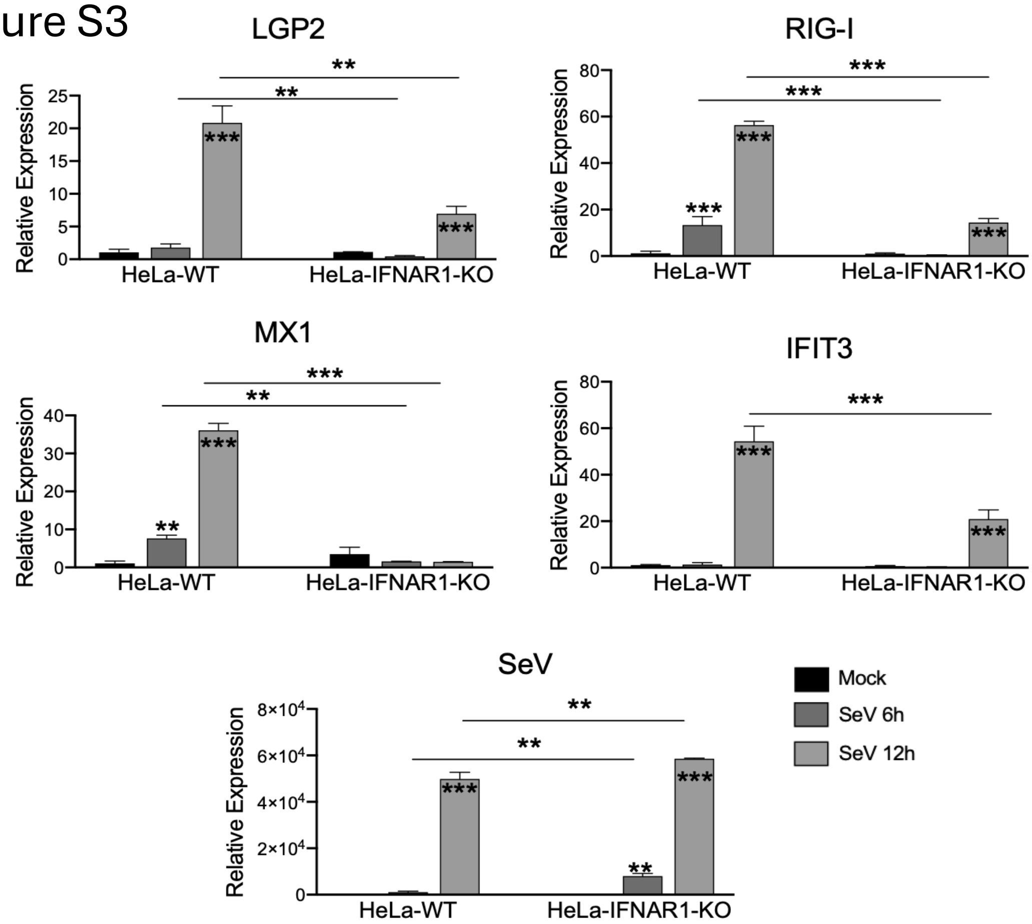

Figure S3. Time-course induction of LGP2 and other ISGs by Sendai virus (SeV) infection as determined by qRT-PCR. HeLa-WT and HeLa-IFNAR1-KO cells seeded at 1.5 × 10^5 cells per well in 12-well plates were mock-infected or infected by SeV at 200 HAU/mL. At 6 and 12 h post infection, cells were harvested for total RNA extraction and cDNA synthesis, followed by qRT-PCR analysis of the expression levels of indicated ISGs and SeV RNA, with 28S rRNA used as the internal control for normalization. Data are from two independent experiments. Error bars represent SD. Asterisks inside/on top of bars represent the significance of the differences in gene expression between that treatment and that cell type's mock treatment. Asterisks on the lines between bars represent the significance of the differences in gene expression between HeLa-WT cells and HeLa IFNAR1-KO cells receiving that treatment. *$p < 0.05$, **$p < 0.01$, ***$p < 0.001$.

Figure S4

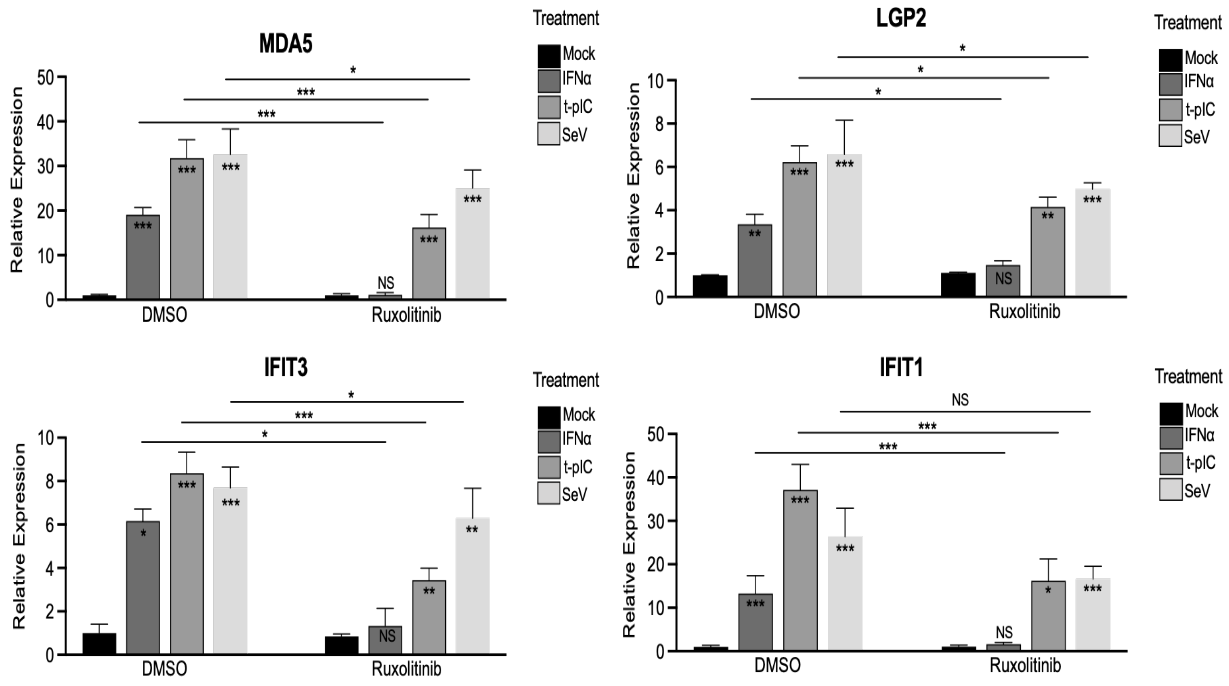

Figure S4. Quantification of Figure 3A immunoblotting data by densitometry analysis. Data are from two independent immunoblotting experiments (normalized to housekeeping loading control) and analyzed by Image Studio Lite (LI-COR Biosciences). Error bars represent SD. Asterisks inside bars represent the significance of the differences in expression between that treatment and that cell type's mock treatment. Asterisks on the lines between bars represent the significance of the differences in expression between the indicated groups. *$p < 0.05$, **$p < 0.01$, ***$p < 0.001$. NS, not significant.

Figure S5

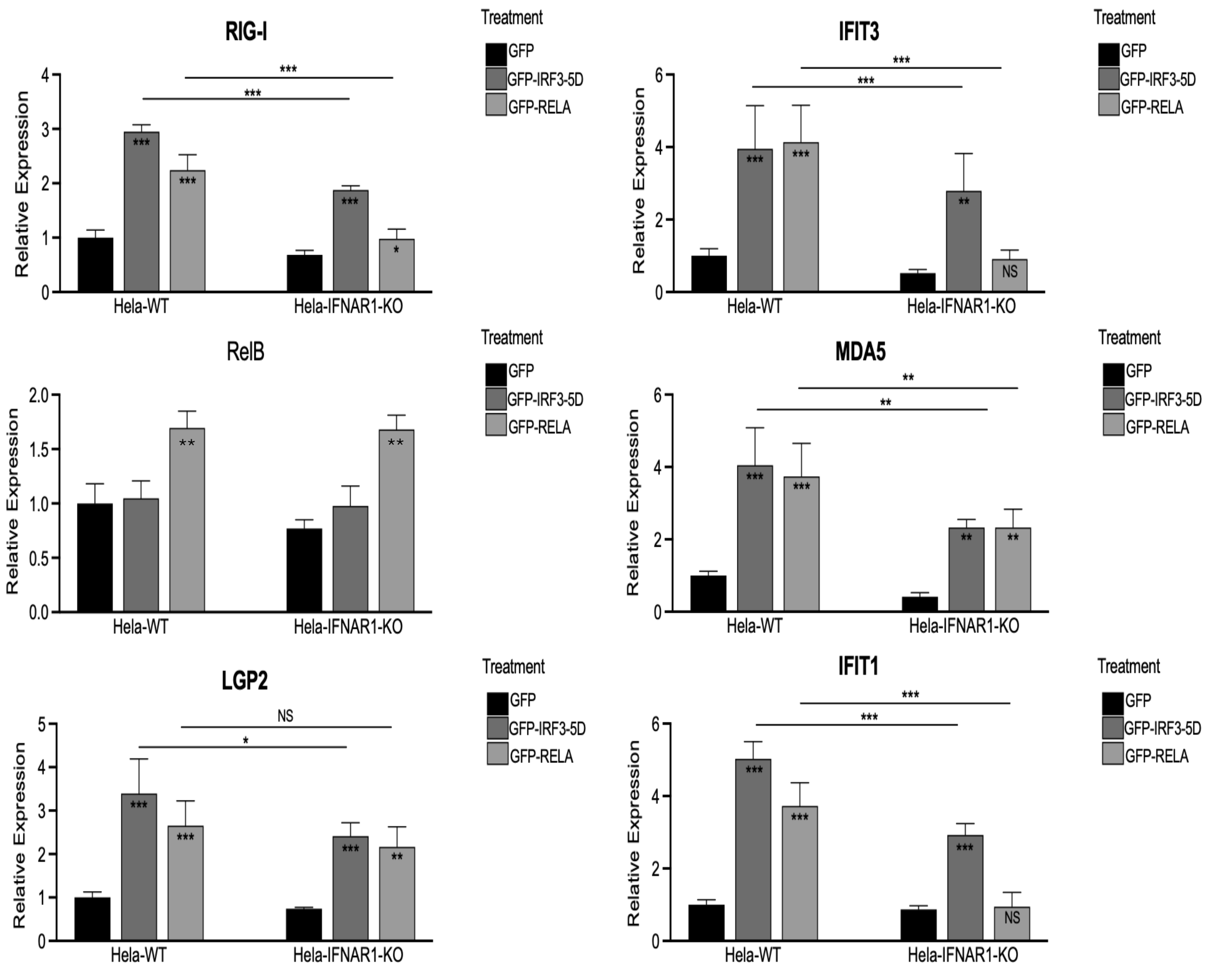

Figure S5. Quantification of Figure 4B immunoblotting data by densitometry analysis. Data are from three independent immunoblotting experiments (normalized to housekeeping loading control) and analyzed by Image Studio Lite (LI-COR Biosciences). Error bars represent SD. Asterisks inside bars represent the significance of the differences in expression between that treatment (transfection of GFP-IRF3-5D or GFP-RELA) and that cell type's mock treatment (transfection of GFP control vector). Asterisks on the lines between bars represent the significance of the differences in expression between the indicated groups. *$p < 0.05$, **$p < 0.01$, ***$p < 0.001$. NS, not significant.
